# Effect of Vegetation Degradation on Soil Nitrogen Components and N-Cycling Enzyme Activities in a Wet Meadow on the Qinghai–Tibetan Plateau

**DOI:** 10.3390/plants14101549

**Published:** 2025-05-21

**Authors:** Wanpeng He, Weiwei Ma, Jianan Du, Wenhua Chang, Guang Li

**Affiliations:** College of Forestry, Gansu Agricultural University, Lanzhou 730070, China; 15117289051@163.com (W.H.); 18419149198@163.com (J.D.); changwh@st.gsau.edu.cn (W.C.); lig@gsau.edu.cn (G.L.)

**Keywords:** vegetation degradation, wet meadow, nitrogen components, enzyme activities

## Abstract

The responses of soil nitrogen component dynamics and enzyme activities to vegetation degradation in wet meadows ecosystems remain unclear. This study employed a combination of field surveys and laboratory experiments to investigate soil nitrogen components and nitrogen cycling enzyme activities under different intensities of vegetation degradation and during the vegetation growth season in a wet meadow on the Qinghai–Tibetan Plateau. The aim was to explore the responses of soil nitrogen components and nitrogen cycling enzyme activities to vegetation degradation and their interrelationships. The results showed that vegetation degradation significantly reduced TN, NH_4_^+^-N, MBN, PRO, and NiR, and increased NO_3_^−^-N, URE, and NR. Soil nitrogen components and enzyme activities exhibited seasonal fluctuations across different degradation levels during the growing season. The Pearson correlation analysis revealed a significant positive correlation between temperature, moisture, nitrogen fractions, and nitrogen cycle-related enzyme activities, as well as between the nitrogen fractions and the enzyme activities themselves. Partial Least Squares Path Modeling (PLS-PM) elucidated the relationships between soil properties and nitrogen components under different degradation levels, explaining 78% of the variance in nitrogen components. Degradation level, growth season, and soil physical properties had indirect associations with nitrogen components, whereas soil enzyme activities exerted a direct positive influence on nitrogen components. Our research revealed the universal impact mechanism of environmental factors, soil characteristics, and vegetation degradation on nitrogen cycling in a wet meadow, thereby making a significant contribution to the restoration and maintenance of functional integrity in alpine wetland ecosystems.

## 1. Introduction

Wetlands serve as critical regulators of global nitrogen (N) cycling, functioning as both major N reservoirs and key mediators of N flux through biotic (e.g., plant uptake, microbial activity) and abiotic (e.g., sedimentation, redox reactions) processes [1,2]. Vegetation plays a central role in these dynamics; wetland plants absorb and immobilize N, while their litter and root exudates drive soil N transformations [3]. However, climate change and anthropogenic disturbances are accelerating vegetation degradation, which alters soil structure, disrupts N sequestration [4], and may destabilize the nitrate (NO_3_^−^-N) to ammonium (NH_4_^+^-N) ratio—ultimately impacting wetland N storage and availability.

Nitrogen is a critical limiting factor for plant growth and ecosystem stability. In wetland ecosystems, plant-available nitrogen primarily comes from soil nitrogen sources, such as mineralization and organic transformations, with soil nitrogen availability directly influenced by changes in its components [5]. Vegetation degradation intensifies soil erosion, leading to significant nitrogen loss and limiting nitrogen accumulation [6,7]. Soil nitrogen availability is a key parameter in plant development within wetland ecosystems. Despite constituting a small fraction of the total nitrogen pool, soil available nitrogen (including MBN, NH_4_^+^-N, NO_3_^−^-N, and TN) is crucial for regulating plant nutrient availability. Nitrogen transformation in these soils is a complex process influenced by both biotic factors (e.g., soil enzymes) and abiotic factors (e.g., soil nutrients, pH), with each transformation stage requiring specific enzymes [8]. The activity levels of nitrogen cycling enzymes (urease (URE), protease (PRO), nitrate reductase (NR), and nitrite reductase (NiR)) reflect soil nitrogen supply and transformation efficiency, indirectly indicating plant nitrogen uptake and utilization [9]. Additionally, vegetation degradation-driven changes in vegetation community, type, management practices, and land use significantly affect soil nitrogen content and its components, inevitably altering soil available nitrogen [10,11]. Understanding the effects of vegetation degradation on soil nitrogen content, particularly the distribution and dynamics of soil nitrogen components and enzyme activities over time, is crucial for predicting ecosystem nitrogen cycling and its implications for global climate change.

The Qinghai–Tibetan Plateau (QTP) is the highest and largest plateau in the world [12], harboring a vast expanse of wetlands, covering an area of approximately 4.9 × 10^4^ km^2^ [13]. Among these, wet meadows occupy about 2.5 × 10^6^ km^2^ (accounting for roughly 35% of the QTP’s total area), serving as a major nitrogen source for the plateau [14,15]. However, over the past several decades, accelerated climate change, the destruction of meadows and vegetation by rodents through tunneling and grazing, and human pressures, such as overgrazing, have led to large-scale vegetation degradation, shrinkage, and transformation in the QTP’s wet grasslands [16,17]. Relevant studies have found that vegetation degradation significantly reduces the vegetation cover, density, height, and biomass of wet meadows on the QTP, exacerbating soil erosion, soil moisture loss, and the depletion of soil nutrients, further impacting ecological functions [18]. However, there is limited research on how vegetation degradation affects soil nitrogen components and nitrogen cycling-related enzyme activities in wet meadows that are degraded on the QTP, as well as the relationships between them. Therefore, understanding the impact of vegetation degradation on soil nitrogen content, especially the distribution and dynamics of soil nitrogen components and enzyme activities in the temporal sequence of vegetation degradation, is of great significance for predicting ecosystem nitrogen cycling and global climate change.

To address these issues, we constructed four levels of vegetation degradation in a wet meadow on the Qinghai–Tibetan Plateau: non-degraded (CK); slightly degraded (SD); moderately degraded (MD); and heavily degraded (HD)). We systematically evaluated the characteristics of changes in soil nitrogen components (i.e., total nitrogen, ammonium nitrogen, nitrate nitrogen, microbial biomass nitrogen) and nitrogen cycling-related enzyme activities (i.e., urease, protease, nitrate reductase, nitrite reductase) during the plant growing season in plots with different degradation levels. Our hypotheses are as follows: (1) Vegetation degradation reduces soil nitrogen content and enzyme activity; (2) Soil nitrogen components and enzyme activities exhibit specific seasonal fluctuations within the growing season and these fluctuations are influenced by the degree of vegetation degradation; and (3) Soil properties (including the degradation level, growing season, and soil physicochemical properties) and soil enzyme activities have a significant combined effect on soil nitrogen components, with soil enzyme activities having a direct positive impact on nitrogen components. By validating these hypotheses, we expect and believe that our research will provide a scientific basis for the ecological restoration and nitrogen management of degraded wetlands under the context of future nitrogen deposition.

## 2. Results

### 2.1. Environmental Factors During the Growing Season

**Figure 1:**
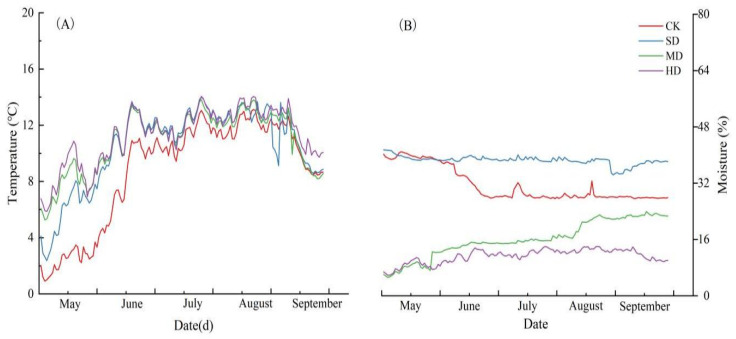
Daily average trends in soil temperature (**A**); and moisture (**B**). CK: non-degraded vegetation; SD: slightly degraded vegetation; MD: moderately degraded vegetation; and HD: heavily degraded vegetation.

Vegetation degradation significantly affected soil temperature and moisture (Figure 1). The study found that the temperatures for CK, SD, MD, and HD were 0.91–13.11 °C, 2.37–14.00 °C, 5.27–13.82 °C, and 5.87–14.05 °C, respectively. The temperature of CK was consistently lower than that of other degradation levels; during the vegetation growing season, the temperatures of all degradation levels showed similar trends. Namely, the temperatures gradually increased from lower temperatures in May to higher temperatures in July and August, followed by a decrease in September (Figure 1A). The moisture contents for each degradation level were 27.57–40.93%, 34.39–41.11%, 5.27–23.96%, and 5.87–14.06%, respectively. SD and HD maintained a relatively stable moisture content throughout the plant growing season; the moisture content of CK continuously decreased throughout the vegetation growing season, showing a sharp decline in June. MD exhibited an opposite trend to CK (Figure 1B).

### 2.2. Spatial and Temporal Variation Characteristics of Soil Nitrogen Components in the Process of Vegetation Degradation

**Figure 2:**
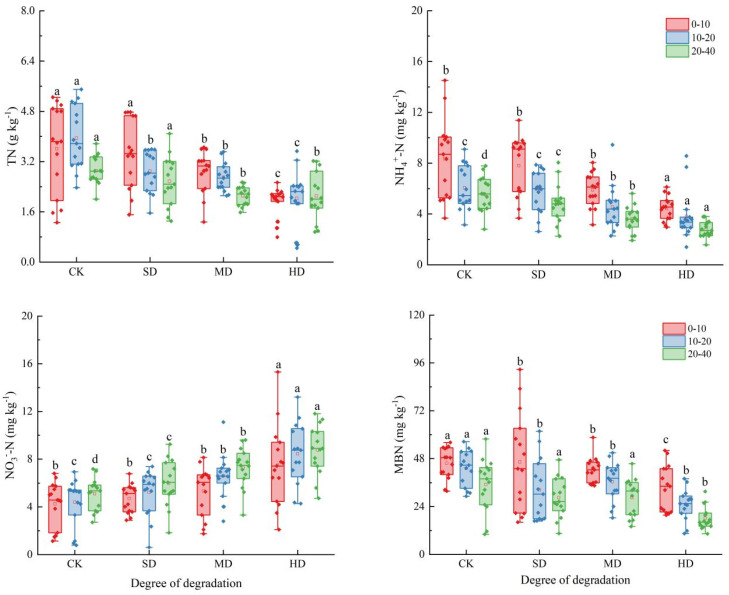
Characteristics of soil nitrogen components in different soil layers during vegetation degradation. CK: non-degraded vegetation; SD: slightly degraded vegetation; MD: moderately degraded vegetation; and HD: heavily degraded vegetation. Different letters indicate significant differences between different degradation levels within the same soil layer (*p* < 0.05).

Vegetation degradation significantly affected the soil nitrogen components in the wet meadow, with a more pronounced impact on the surface soil layers (0–10 and 10–20 cm) compared to the deeper soil layer (20–40 cm) (Figure 2). The contents of soil TN, NH_4_^+^-N, NO_3_^−^-N, and MBN were 1.93–3.4 g kg^−1^, 2.78–8.34 mg kg^−1^, 4.26–8.76 mg kg^−1^, and 19.45–56.89 mg kg^−1^, respectively. With the intensification of degradation, compared to the CK, the other degradation levels showed reductions in TN by 13.91%, 26.43%, and 41.02%, reductions in NH_4_^+^-N by 7.92%, 29.98%, and 44.74%, and reductions in MBN by 21.31%, 20.65%, and 41.88%, respectively, while NO_3_^−^-N increased by 13.33%, 27.83%, and 44.24%, respectively. The soil nitrogen components exhibited clear vertical distribution characteristics, with the TN, NH_4_^+^-N, and MBN contents in the 0–10 and 10–20 cm soil layers of the CK plot being significantly higher than those of the other degradation levels, whereas the NO_3_^−^-N content was lower than that of the other degradation levels. Vegetation degradation and soil layer changes had a highly significant impact on nitrogen components (*p* < 0.01); there was also a significant interaction effect between vegetation degradation and the soil layer on TN (*p* < 0.05) (Table 1).

**Figure 3:**
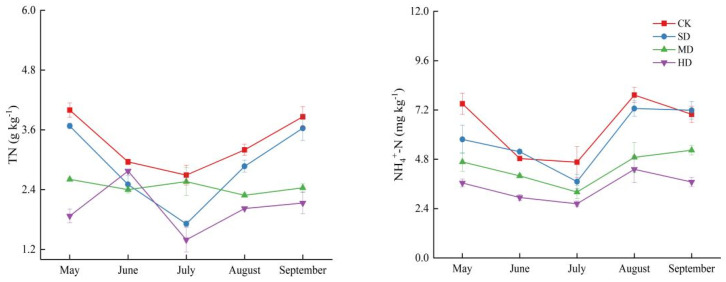

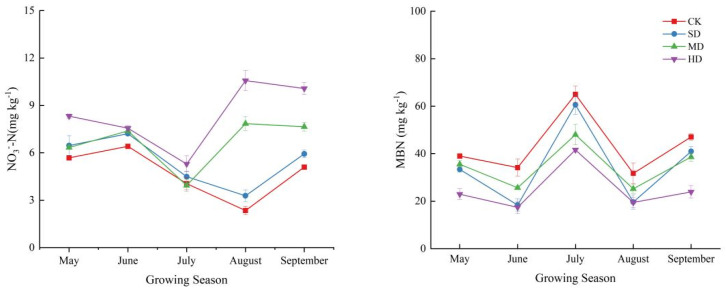
Characteristics of soil nitrogen components during the vegetation growth season in the process of vegetation degradation. CK: non-degraded vegetation; SD: slightly degraded vegetation; MD: moderately degraded vegetation; and HD: heavily degraded vegetation.

The soil nitrogen components in the wet meadow during the plant growing season responded differently to vegetation degradation; the soil N components exhibited significant seasonal fluctuations (Figure 3). The study found that the soil TN in CK, SD, and HD showed a trend of initially decreasing and then increasing during the vegetation growing season, reaching the lowest values in July (2.69, 1.71, and 1.39 g kg^−1^). The seasonal changes in NH_4_^+^-N at each degradation level were generally the same, with the minimum values occurring in July (4.66, 3.72, 3.21, and 2.64 mg kg^−1^). The NO_3_^−^-N fluctuated significantly throughout the growing season, with CK and SD showing a trend of initially increasing, then decreasing, and finally increasing again, with the minimum values occurring in July (2.35 and 3.28 mg kg^−1^). The changes in MD and HD were opposite to those in CK and SD, with the maximum values occurring in August (7.84 and 10.57 mg kg^−1^). MBN also fluctuated during the growing season, with a significant increase from June to July, reaching peak values in July (64.97, 60.58, 48.03, and 41.63 mg kg^−1^). Vegetation degradation, the plant growing season, and their interaction significantly affected the soil nitrogen components (*p* < 0.01) (Table 1).

### 2.3. Spatial and Temporal Variation Characteristics of Soil Enzyme Activities During Vegetation Degradation

**Figure 4:**
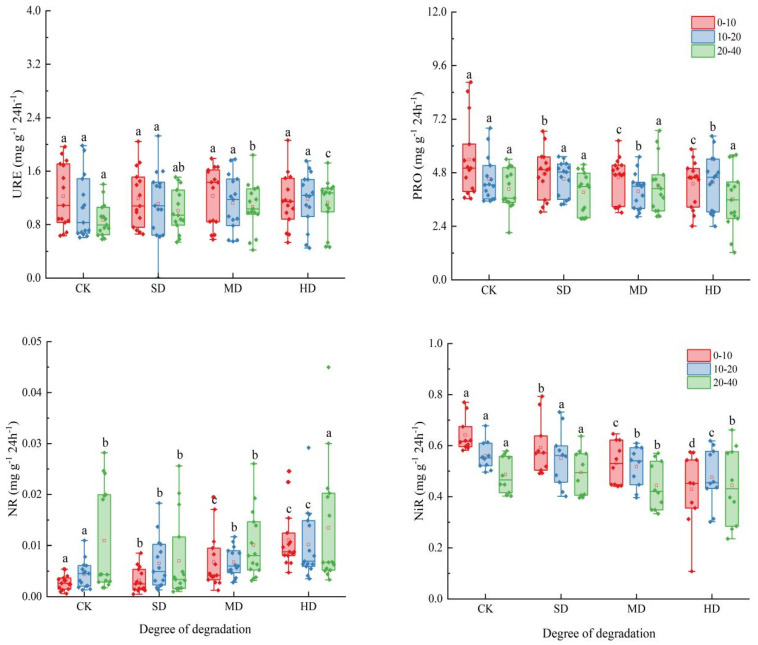
Soil enzyme activities in different soil layers during vegetation degradation. CK: non-degraded vegetation; SD: slightly degraded vegetation; MD: moderately degraded vegetation; and HD: heavily degraded vegetation. Different letters indicate significant differences between different degradation levels within the same soil layer (*p* < 0.05).

Significant differences in soil nitrogen cycling enzyme activities were observed in soil profiles under different degrees of vegetation degradation (Figure 4). This study found that the activities of nitrogen cycling enzymes were 0.87–1.23 mg g^−1^ 24 h^−1^, 3.61–5.38 mg g^−1^ 24 h^−1^, 0.0028–0.0135 mg g^−1^ 24 h^−1^, and 0.43–0.64 mg g^−1^ 24 h^−1^, respectively. Vegetation degradation increased soil URE and NR while decreasing PRO and NiR. Compared to CK, URE and NR in SD, MD, and HD increased by 5.20%, 8.49%, 10.06% and 7.85%, 21.26%, 107.33%, while PRO and NiR decreased by 5.50%, 8.39%, 12.05% and 3.09%, 11.65%, 20.13%. The study also found that soil enzyme activities exhibited significant vertical distribution characteristics; specifically, URE, PRO, and NiR decreased with increasing soil depth, while NR increased. The impact on enzyme activities in the surface layer (0–10 cm) was particularly significant, with significant differences in PRO, NR, and NiR among CK, MD, and HD in the 0–10 cm soil layer. Vegetation degradation, the soil layer, and the interaction between vegetation degradation and the soil layer significantly affected soil enzyme activities (*p* < 0.01) (Table 1).

**Figure 5:**
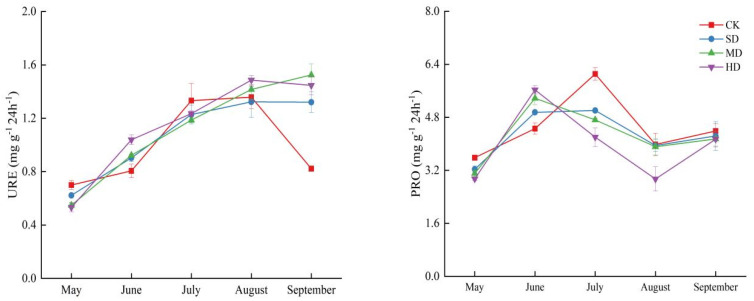

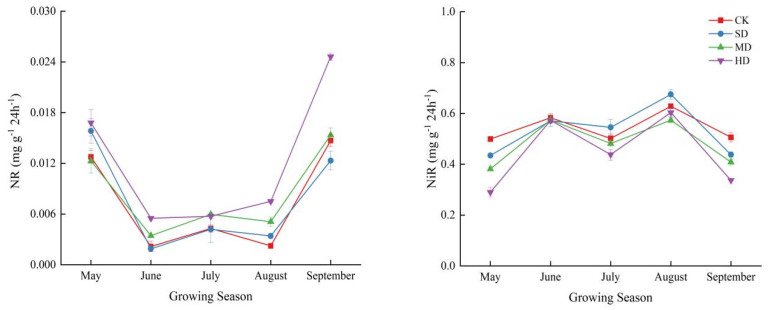
Soil enzyme activities during the vegetation growing season in the process of vegetation degradation. CK: non-degraded vegetation; SD: slightly degraded vegetation; MD: moderately degraded vegetation; and HD: heavily degraded vegetation.

Soil nitrogen cycling enzyme activities exhibited fluctuating changes at different plant growth stages under the influence of vegetation degradation (Figure 5). The study found that soil URE activity at all four degradation levels increased during the vegetation growth season, reaching its maximum in August (1.36, 1.32, 1.49 mg g^−1^ 24 h^−1^) for all levels except MD. The seasonal variation of PRO at each degradation level showed the trend of an initial increase, followed by a decrease, and then another increase. However, the maximum PRO activity for SD, MD, and HD occurred in June (5.38, 5.63 mg g^−1^ 24 h^−1^); for CK, the maximum was observed in July (5.01 mg g^−1^ 24 h^−1^). NR and NiR exhibited significant seasonal fluctuations; the trends of change were generally consistent across all degradation levels. NR showed a pattern of initial decrease, followed by an increase, then another decrease, and finally another increase. In contrast, NiR exhibited a trend opposite to that of NR. The minimum value of NR appeared in June (0.0022, 0.0019, 0.034, 0.0055 mg g^−1^ 24 h^−1^) and NiR reached its peak in August (0.63, 0.67, 0.57, 0.60 mg g^−1^ 24 h^−1^). Additionally, vegetation degradation, the growth season, and the interaction between vegetation degradation and growth season significantly affected soil nitrogen-related enzymes (*p* < 0.01) (Table 1).

### 2.4. Correlation Between Soil Nitrogen Components and Enzyme Activity Under Vegetation Degradation

**Figure 6:**
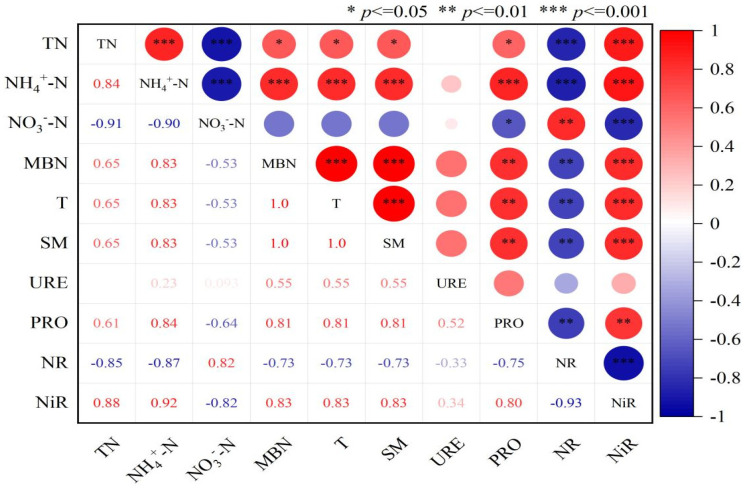
Correlation between soil nitrogen components and enzyme activity.

The Pearson’s correlation analysis indicated that soil nitrogen components were significantly positively correlated with enzyme activities (Figure 6). Soil temperature and moisture were significantly positively correlated with TN, MBN, NH_4_^+^-N, PRO, and NiR (*p* < 0.01); they were significantly negatively correlated with NR and NO_3_^−^-N (*p* < 0.01). Among the soil nitrogen components, TN, NH_4_^+^-N, and MBN showed extremely significant positive correlations with each other (*p* < 0.01). Among the soil enzyme activities, URE, PRO, and NiR exhibited significant positive correlations with each other (*p* < 0.05). Except for NO_3_^−^-N and NR, other enzyme activities also showed significant positive correlations with nitrogen components (*p* < 0.05).

### 2.5. Relationship Between Environmental Variables and Soil N Components

**Figure 7:**
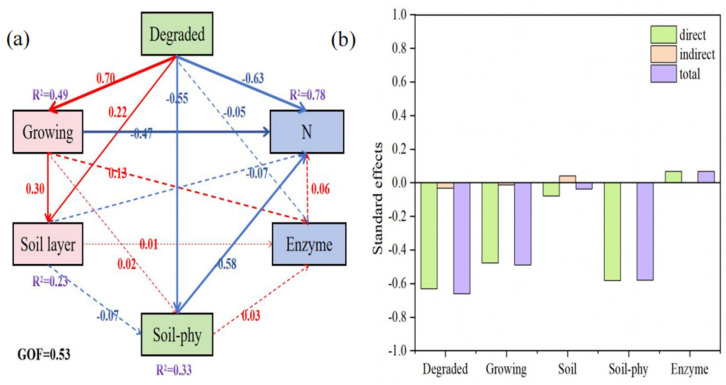
The impact of soil properties and enzyme activity on N components under different degrees of vegetation degradation. The Partial Least Squares Path Model (PLS-PM) shows the impact of land use on N emissions (**a**); and the standardized total impact of environmental variables obtained from PLS-PM (**b**). The number next to the arrow represents the standardized path coefficient and the width of the arrow is proportional to the strength of the association. The red arrow represents a positive correlation, while the blue arrow represents a negative correlation. The R^2^ value represents the variance of the variable that the model can explain. The physical properties of soil (soil_phy) are soil physical properties (Soil-phy) and are represented by the soil temperature (ST); soil moisture content (STW); bulk density (BD); and field water-holding capacity (FC). The growing season (Growing) is represented by the months of May, June, July, August, and September; the soil layers are divided into 0—10, 0—20, and 20—40 cm; and the enzyme activity is indicated by urease (URE), proteinase (PRO), nitrate reductase (NR), and nitrite reductase (NiR).

In order to quantify the relationship between soil properties and N components under different land use patterns, we conducted further analysis using PLS-PM. Based on the selected variable, PLS-PM explains the variance of 78% of the N components (Figure 7a). The results indicate that the degree of degradation, growing season, and soil physical properties had a relatively high overall impact on N components (−0.66, −0.49, and −0.58) (Figure 7b). Among them, vegetation degradation had a significant indirect effect on N components through its impact on soil layers; however, the direct effect on N components was not significant (Figure 7a). Soil enzyme activity had a direct positive effect on nitrogen components.

## 3. Discussion

### 3.1. Response of Soil Nitrogen Components to Vegetation Degradation

Soil nitrogen, as a key factor directly affecting plant growth, exhibits variations in its component content and forms that are direct indicators of soil fertility. The diversity of soil texture, the chemical characteristics of organic carbon sources, and the complexity of microbial community activities all exert varying degrees of regulatory effects on the content and spatial distribution of nitrogen components in the soil [19]. Vegetation degradation can, to some extent, have positive effects on temperature and humidity conditions, water cycling processes, and the decomposition rate of litter, while plant root exudates can also effectively promote the transformation processes of soil nitrogen [20,21]. The results of this study indicate that the response patterns of soil nitrogen components to different degrees of vegetation degradation exhibit significant differences. Vegetation degradation significantly reduced the contents of soil TN, NH_4_^+^-N, and MBN, while increasing the content of NO_3_^−^-N, which is consistent with research findings from degraded areas such as the Amazon rainforest [22] and the African Sahel region [23]. On the one hand, this is because vegetation degradation leads to increased soil erosion due to surface exposure, especially in MD and HD with lower vegetation cover (Table 2), resulting in severe nutrient loss [24]. On the other hand, vegetation degradation reduces the input of plant residues, thereby decreasing the input of soil organic matter and limiting the availability of nitrogen sources [25]. Additionally, vegetation degradation alters rhizosphere dynamics, leading to a reduction in root exudates, which in turn lowers the activity of nitrogen cycling-related microorganisms, further affecting nitrogen mineralization and immobilization processes [26]. Conversely, the increase in NO_3_^−^-N content may be attributed to enhanced nitrification processes. Vegetation degradation results in a more aerobic soil environment, favoring nitrifying bacteria in converting more NH_4_ to NO_3_. Moreover, in degraded vegetation areas, the reduced uptake of NO_3_ by plants facilitates its accumulation in the soil [27]. The soil N content in degraded plots is not only related to vegetation, microbial activity, and soil erosion but may also be associated with soil temperature, moisture, and enzyme activity. The significant positive correlations between soil N content and T, SM, and enzyme activity confirm the aforementioned hypotheses (Figure 6).

The growing season is the most critical stage in the life cycle of plants, exerting a significant direct impact on the processes of nitrogen fixation and transformation in ecosystems [28]. Relevant studies [29] have shown that nitrogen dynamics during the vegetation growing season exhibit pronounced seasonal variations; climate warming and changes in precipitation patterns significantly affect nitrogen mineralization and immobilization processes by altering the length and intensity of the growing season. Our research reveals the complex seasonal dynamics of soil nitrogen components at different levels of degradation (Figure 3). It was observed that soil TN decreased initially and then increased during the vegetation growing season, reaching its lowest point in July, which is similar to that of previous findings [19,30]. This is because rainfall-leaching in the early stages of vegetation growth leads to nitrogen loss; the increased demand for nitrogen by growing vegetation continuously depletes nitrogen. Additionally, related studies indicate that as the growing season progresses, the demand for nitrogen by plants weakens; the decomposition of plant residues and organic matter contributes to the subsequent increase in TN [30]. At all degradation levels, the seasonal variation in NH_4_^+^-N content exhibited a similar pattern: an initial decrease, followed by an increase and then a final decrease, with the lowest value occurring in July. This is inconsistent with existing research results [31], which may have been due to the higher soil pH value (Table 3) and elevated temperature (Figure 1A) enhancing, in our study area, the activity of nitrifying bacteria. This acceleration of the conversion of NH_4_^+^-N to NO_3_^−^-N (Figure 2) resulted in an increased nitrification rate and a higher demand for nitrogen by plants, leading to an initial reduction in NH_4_^+^-N. The subsequent increase is likely due to reduced plant uptake and enhanced microbial activity under the high temperature and humidity conditions of summer, which increases the mineralization of organic nitrogen, followed by a final decrease due to renewed plant nitrogen uptake and continuous nitrification. The seasonal dynamics of NO_3_^−^-N content in CK and SD showed an initial decline, followed by an increase and then a final decrease, with the minimum value in July. However, the changes in MD and HD were opposite to those in CK and SD, with the minimum value occurring in August. Studies on degraded alpine wet meadows have found that soil NO_3_^−^-N increases continuously with the growth stages of vegetation; NO_3_^−^-N levels in summer are significantly higher than in spring, without a distinct peak [32]. This is inconsistent with the results of this study, which may be due to differences in geographical location and climate. In this study area, the CK and SD treatments have better vegetation cover and more vigorous root activity, especially during the peak of the growing season (such as in August), enabling them to absorb more NO_3_^−^-N. Additionally, microbial activity is relatively high; the balance between nitrification and denitrification may be more favorable, aiding in the transformation and consumption of NO_3_^−^-N. In contrast, MD and HD have less vegetation cover and relatively lower NO_3_^−^-N uptake. Their microbial community structure may have changed due to degradation, with enhanced nitrification and reduced denitrification leading to the continuous accumulation of NO_3_^−^-N [33]. Consistent with previous research results [30], MBN also showed fluctuating changes during the growing season, peaking in July. This may have been due to the increased microbial activity, optimal soil temperature and moisture conditions, and enhanced availability of organic substrates, leading to an increase in microbial biomass. Subsequently, the increased microbial turnover rate, and substantial nitrogen uptake by plants during their peak growth period decrease the nitrogen sources available to microorganisms [34].

### 3.2. Response of Soil Enzyme Activity to Vegetation Degradation

Soil enzyme activity refers to the catalytic capacity of various enzymes in the soil that participate in, and promote, soil development and fertility formation [35]. The level of enzyme activity not only directly affects soil biochemical processes but also reflects changes in the soil microenvironment [36]. Urease, protease, nitrate reductase, and nitrite reductase are closely related to the soil’s nitrogen supply capacity and the transformation of nitrogen in the soil [9,37]. This study found that vegetation degradation increased the activities of URE and NR, while decreasing the activities of PRO and NiR, consistent with previous research results [38]. The main reasons for this are as follows: the increase in URE activity may be due to vegetation degradation leading to an increase in urea content in the soil, which in turn alters the structure of the soil microbial community, thereby enhancing the relative abundance of URE-producing bacteria. This also further promotes the rate of nitrogen transformation [39]. Vegetation degradation alters the redox conditions within the soil, accompanied by changes in soil nitrogen, especially the accumulation of nitrate nitrogen. NR, as a key enzyme in the assimilation process of nitrate nitrogen, its increased activity further accelerates the reduction process of soil nitrate nitrogen [40,41]. The decrease in PRO and NiR activities may be due to the obstruction of the mineralization process of organic nitrogen and the weakening of the nitrite nitrogen reduction process, affecting N transformation and cycling [42]. Moreover, soil enzyme activity exhibits distinct vertical distribution characteristics. In the 0–10 cm soil layer, there are significant differences in soil enzyme activity among different degradation levels (CK, MD, SD). Since the surface soil is directly influenced by vegetation cover and root activity, it is usually rich in organic matter and nitrogen, providing ample substrates for enzymes. The changes in soil environment caused by vegetation degradation are most pronounced in this layer, thereby leading to significant differences in enzyme activity [43]. This indicates that vegetation degradation has a significant impact on soil profile characteristics, especially the enzyme activity in the surface soil, which is more sensitive.

Vegetation degradation significantly affected the activities of nitrogen-related enzymes in degraded wetland soils; this impact varied significantly across different growing seasons (Figure 4). Consistent with previous research results [44], the activities of URE, PRO, NR, and NiR in soils at various degradation levels increased as the vegetation growing season progressed. This is because, as the vegetation growing season advances, soil microbial activity intensifies, accelerating the decomposition of organic matter and increasing URE activity. Additionally, the increase in plant residues and root exudates provides more substrates, leading to elevated PRO activity. Furthermore, enhanced root activity and altered soil redox processes with vegetation growth also promote the increase in NR and NiR activities [45]. This study also found that, except for MD, URE activity at all other degradation levels showed significant growth during different vegetation growth stages, with the highest URE activity in August, consistent with existing research results [46]. This is due to the increased root exudates and litter as vegetation grows, providing more organic nitrogen sources and promoting the production and activity of URE [47]. Additionally, August is the peak period of vegetation growth and is characterized by high temperature and humidity conditions (Figure 1), resulting in the most vigorous microbial activity and maximal URE activity [48]. The absence of peak URE activity in August at the MD level may be related to its specific microbial community structure, causing the peak of URE activity to occur earlier or later [49]. PRO activity at various degradation levels exhibited a trend of initial increase, followed by a decrease, and then another increase; however, the timing of the maximum values differed among degradation levels. The maximum PRO values for SD, MD, and HD appeared in June; for CK, the maximum occurred in July, which is generally consistent with previous research results [50,51]. This may be because June and July are critical periods for vegetation growth; differences in the content and distribution of proteinaceous organic matter in the soil, as well as the functionality and activity of microbial communities at different degradation levels, affect the production and degradation of PRO, leading to different timings of PRO activity peaks. CK, with higher organic matter quality and better vegetation cover, may delay the PRO activity peak until July. NR and NiR showed distinct seasonal fluctuations at different degradation levels. NR exhibited a trend of initial decrease, followed by an increase, then a decrease, and another increase. NiR showed an opposite trend to NR; the minimum NR value occurred in June and NiR peaked in August. NR is involved in the reduction in nitrate nitrogen; its activity is influenced by the soil nitrate nitrogen content [52]. In June, when vegetation growth is vigorous, more soil nitrate nitrogen is consumed, leading to a decrease in NR activity. NiR is involved in the reduction in nitrite; its activity peak in August may be related to the accumulation of nitrite in the soil at this time [53]. Seasonal changes in environmental factors, such as soil temperature and humidity, also affect the activities of NR and NiR (Figure 6). The seasonal variations in URE and PRO activities reflect the transformation processes of soil organic nitrogen, while the fluctuations in NR and NiR activities reveal the dynamic changes in soil inorganic nitrogen cycling.

### 3.3. Relationships Between Soil Nitrogen, Enzyme Activities, and Environmental Factors

Through the correlation analysis between soil nitrogen content and enzyme activity (Figure 6), this study revealed the complex relationships between soil environmental factors (temperature and moisture) and nitrogen components with enzyme activity. The results show that soil temperature and moisture were significantly positively correlated with TN, MBN, NH_4_^+^-N, PRO, and NiR (*p* < 0.01), and were significantly negatively correlated with NR and NO_3_^−^-N (*p* < 0.01). This indicates that soil temperature and moisture are key factors affecting soil nitrogen transformation and enzyme activity. Higher soil temperature and moisture favor the accumulation of TN, MBN, and NH_4_^+^-N, likely because these conditions promote microbial activity and organic matter decomposition, thereby increasing nitrogen mineralization [54], and also enhancing the denitrification of nitrate nitrogen, leading to a decrease in NO_3_^−^-N. The highly significant positive correlations among TN, NH_4_^+^-N, and MBN in soil nitrogen components indicate their close relationships in the soil nitrogen cycling process. An increase in TN not only promotes ammonification to increase NH_4_^+^-N but also leads to the accumulation of MBN. Additionally, the increase in MBN promotes the mineralization of organic nitrogen [51,55]. The positive correlations between soil enzyme activities reflect their synergistic roles in the soil nitrogen transformation process. Furthermore, except for NO_3_^−^-N and NR, other enzyme activities were also significantly positively correlated with nitrogen components (*p* < 0.05), indicating a close interaction between soil enzyme activity and nitrogen components. The increase in enzyme activity promotes the mineralization and transformation of organic nitrogen, thereby increasing the contents of TN, MBN, and NH_4_^+^-N [56]. The negative correlation between NO_3_^−^-N and NR: A decrease in NR activity may lead to the accumulation of nitrate nitrogen, while a reduction in NO_3_^−^-N may inhibit NR activity [57].

To quantify the relationships between soil properties and nitrogen components under different land use patterns, we further employed a Partial Least Squares Path Model (PLS-PM) for the analysis. Based on the selected variables, the PLS-PM explained 78% of the variance in nitrogen components (Figure 7a), which indicated that the selected variables have a good explanatory power for the variation in nitrogen components; it also validated the rationality of the selected variables and the effectiveness of the model. The study found that the degree of degradation, growing season, and soil physical properties had relatively high overall impacts on nitrogen components (−0.66, −0.49, and −0.58) (Figure 7b). These findings reveal the important roles of environmental factors and soil properties in regulating the nitrogen cycle. The indirect effect of vegetation degradation on nitrogen components was significant, primarily through its impact on the soil layer; however, its direct effect on nitrogen components was not significant (Figure 7a). This suggests that vegetation degradation indirectly affects nitrogen transformation and accumulation by altering the soil structure and hydrological conditions [52]. For example, degradation may lead to thinning of the soil layer, affecting nitrogen leaching and fixation [58]. The impacts of the growing season on nitrogen components indicates that seasonal changes play a crucial role in the nitrogen cycle. During the growing season, the increased demand for nitrogen by plants may lead to a decrease in nitrogen components in the soil [59]. Additionally, temperature and precipitation variations in different seasons can also affect microbial activity and enzyme activity, thereby influencing nitrogen transformation [60]. The negative impact of soil physical properties (such as texture, porosity, etc.) on nitrogen components indicates that the physical structure of the soil plays an important role in nitrogen retention and migration. Good soil physical properties help to maintain the stability and effectiveness of nitrogen [61]. The study also found that soil enzyme activity had a direct positive effect on nitrogen components. This result is consistent with previous studies, indicating that soil enzymes play a key role in the mineralization, transformation, and fixation of nitrogen [62]. Increased enzyme activity can accelerate the decomposition of organic nitrogen and enhance the bioavailability of nitrogen, thereby promoting plant growth and soil nitrogen cycling [63].

Despite the valuable insights provided by this study, we acknowledge several limitations that may have influenced the interpretation and generalizability of our results. First, the research was conducted within a specific geographic region, which may limit the universality of our findings. Second, the experimental design did not account for the variability of certain environmental factors (such as rainfall and soil type), potentially leading to an incomplete understanding of the interactions between the vegetation and soil. Lastly, the study lacks an in-depth examination of microbial community structure, which may hinder our ability to fully comprehend how vegetation degradation affects the functional and structural stability of the soil ecosystem. Future research could enhance the reliability and generalizability of the results by conducting replicate experiments across multiple geographic regions. Additionally, incorporating a broader range of environmental factors into comprehensive study designs would facilitate a more holistic understanding of the complex interactions between vegetation growth and soil nitrogen dynamics. Finally, detailed analyses of soil microbial communities and structures are essential to comprehensively assess the impacts of vegetation degradation on soil ecosystems. Employing long-term monitoring methods to capture the effects of seasonal and annual variations on soil nitrogen dynamics would provide scientific basis for the restoration and management of degraded vegetation.

## 4. Materials and Methods

### 4.1. Study Area

**Figure 8:**
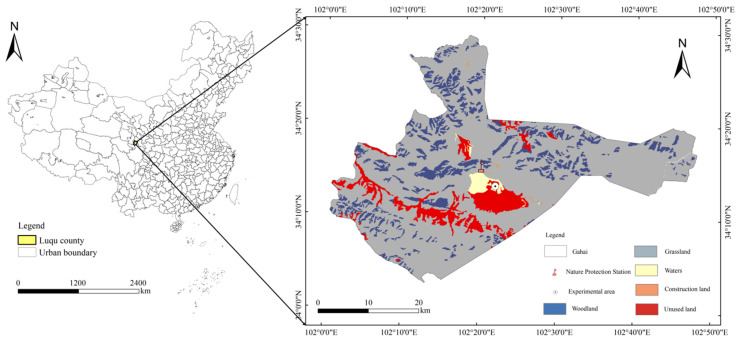
Location map of the study area.

The study site is located in the Gahai-Zecha International Nature Reserve in the Gannan Tibetan Autonomous Prefecture, Gansu Province, China (34°16′ N, 102°26′ E) (Figure 8). The dominant vegetation species in the study area include *Carex meyeriana*, *Thalictrum aquilegifolium*, *Artemisia subulata*, *Potentilla chinensis*, *Polygonum viviparum* L., and *Potentilla anserina*. The region belongs to the Qinghai–Tibetan Plateau zone, characterized by a cold and humid climate, with an average annual temperature of 2.9 °C, an annual evaporation rate of 1150.5 mm, and no absolute frost-free period. The rainy season is mainly concentrated from July to September, with an average annual precipitation of 593 mm. The primary soil type in this area is meadow soil [18].

### 4.2. Materials and Experimental Design

**Figure 9:**
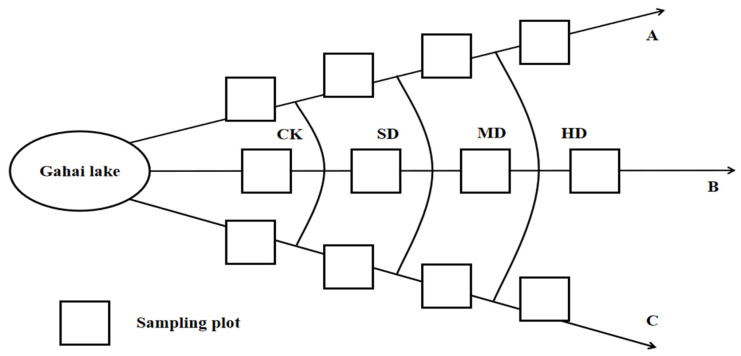
Sampling point: A, B, and C represent three transects. CK: non-degraded vegetation; SD: slightly degraded vegetation; MD: moderately degraded vegetation; HD: heavily degraded vegetation.

Based on the research work of our team, we adopted a spatial sequence instead of a temporal sequence approach. Focusing on the currently visible pristine wetlands during the growing season, we extended outward to select areas with relatively gentle slopes and consistent aspects. According to characteristics, such as vegetation composition, vegetation coverage, the composition of dominant species, and above-ground biomass (Table 2), we established four vegetation degradation gradient plots: non-degraded (CK); slightly degraded (SD); moderately degraded (MD); and heavily degraded (HD) (Figure 9) [15,53]. Each degradation gradient plot measured 10 m × 10 m (100 m^2^) and was replicated three times, with an interval of more than 10 m between replicates [64,65]. The plots were fenced to prevent interference from humans and livestock. The plots were set up in 2015; detailed information about the plots can be found in our team’s previous reports (Table 3).

### 4.3. Soil Sampling and Analysis

In 2019, soil samples were collected within different vegetation degradation gradient plots according to the plant growing season (May to September). Soil samples were taken using a soil auger at each study plot following the ‘S’ five-point method, with separate sampling at 0–10 cm, 10–20 cm, and 20–40 cm depths. Soil from the same layer was mixed to form a composite sample, with plant roots and stones removed. Each soil sample was placed in a sealed bag with four replicates and transported in a cooler with ice packs to maintain low temperatures. Upon returning to the laboratory, part of each soil sample was used for the determination of soil moisture content and microbial biomass, while the other part was sieved through a 1 mm sieve and stored in sealed bags for the analysis of nitrogen components and enzyme activity indices.

Specifically, total nitrogen (TN) was determined using the semi-micro Kjeldahl method [66]; 0.5 g of air-dried soil was weighed and the sample was moistened with 1 mL of distilled water; 8 mL of H_2_SO_4_ was added and the mixture was mixed well. A bent-neck funnel was placed on top and the mixture was digested in a digestion furnace at 400 °C. When white smoke appeared, 10 mL of H_2_O_2_ was added. Digestion was continued and hydrogen peroxide was added every 20 min; this process was repeated 3–5 times. Once the solution became colorless or white, it was removed and cooled, washed in a 100 mL volumetric flask, diluted to volume with water, and mixed well. A 5 mL pipette of the solution was placed into a digestion tube, distilled for 4 min using a Kjeldahl nitrogen analyzer, and finally titrated with standard acid until the solution changed from blue green to pink and remained unchanged. The volume of standard acid consumed during the titration was recorded. Ammonium nitrogen (NH_4_^+^-N) and nitrate nitrogen (NO_3_^−^-N) were measured using the MgO–Denigès alloy distillation method [67], 10 g of the soil sample that had passed through a 2 mm sieve was weighed, 50 mL of KCl was added, and the solution was shaken on a shaker for 1 h. The mixture was allowed to stand and clarify for 30 min. A 10 mL pipette of the clarified supernatant was added into a Kjeldahl tube; 0.5 g of MgO was added and the solution was distilled for 3 min using a Kjeldahl nitrogen analyzer. The distillate was collected in a condenser with 5 mL of boric acid solution for absorption and titrated with a standard H_2_SO_4_ solution. After the above distillation was complete, 1 mL of sulfamic acid was added to the solution; it was shaken for 5 s and 0.5 g of Devarda’s alloy powder was added. The solution was distilled for another 3 min, and the condensate was collected in 5 mL of boric acid solution for absorption, followed by titration with a standard H_2_SO_4_ solution. Microbial nitrogen (MBN) content was determined using the chloroform fumigation–K_2_SO_4_ extraction method [68]; 5 g of the fresh soil sample that had passed through a 2 mm sieve into a 50 mL beaker was weighed. A small beaker containing dilute NaOH (to absorb CO_2_) and another small beaker of water were placed at the bottom of a vacuum desiccator. In the upper part of the desiccator, a small beaker containing ethanol-free chloroform was placed, with several clean small glass beads added to prevent bumping. The desiccator was sealed with Vaseline and it was evacuated using a vacuum pump until the chloroform boiled. Pumping was continued for 5–10 min. The valve of the vacuum desiccator was closed and incubated in the dark at 25 °C for 24 h. After fumigation, the desiccator valve was opened, the beaker was removed, and the fume hood was opened to eliminate residual chloroform in the soil. Simultaneously, three identical portions of soil were weighed for use as controls without the fumigation treatment. The fumigated soil samples and the unfumigated control samples were transferred to conical flasks. Then, 25 mL of K_2_SO_4_ solution was added and the solution was shaken for 30 min and then filtered. The extract was pipetted and its absorbance was measured at 280 nm using a UV spectrophotometer. Soil urease (URE) activity was assessed using the urea colorimetric method [69]; 5 g of the soil sample was added into a 50 mL conical flask with 1 mL of toluene and shaken evenly. After 15 min, 10 mL of 10% urea solution and 20 mL of pH 6.7 citrate buffer solution were added, the mixture was mixed well and incubated in a constant temperature incubator at 37 °C for 24 h. After incubation, the mixture was filtered and 1 mL of the filtrate was added to a 50 mL volumetric flask with 4 mL of sodium phenolate solution and 3 mL of sodium hypochlorite solution; the mixture was shaken well as each reagent was added. After 20 min, the color developed; then, the volume was adjusted to the mark. The absorbance of the solution at 578 nm was measured using a spectrophotometer within 1 h. Protease (PRO) activity was measured using the casein colorimetric method [70]; 2 g of fresh soil that had passed through a 2 mm sieve was added and the mixture was placed in a 50 mL volumetric flask; 1.00 mL of toluene was added (as a bacteriostatic agent to inhibit microbial activity) and 20 mL of 1% casein solution prepared with a pH 7.4 phosphate buffer solution was added. The mixture was incubated in a constant temperature incubator at 30 °C for 24 h. After incubation, the contents of the flask were filtered and 5 mL of the filtrate was placed in a test tube with 0.5 mL of 0.1N sulfuric acid and 3 mL of 20% sodium sulfate to precipitate the proteins. The mixture was filtrated into a 50 mL volumetric flask; 1 mL of 2% ninhydrin solution was added. The mixture was shaken and heated in a boiling water bath for 10 min. The resulting colored solution was diluted with distilled water to the mark. Finally, the absorbance was measured at 560 nm using a spectrophotometer. Soil nitrate reductase (NR) and nitrite reductase (NiR) activities were determined using the benzene sulfonic acid–acetic acid-α-naphthylamine colorimetric method [71]. Then, 1 g of the soil sample was added to 0.02 g of CaCO_3_ and 2 mL of 0.2% NaNO_2_ solution. Then, 2 mL of a 1% glucose solution was added and the solution was supplemented with 5 mL of distilled water to a total volume of 10 mL. The mixture was sealed and incubated at 30 °C for 24 h. After incubation, the contents were washed into a 100 mL conical flask using 50 mL of distilled water and 1 mL of saturated potassium aluminum sulfate solution. The solution was shaken well and then filtered. Then, 1 mL of the filtrate was placed in a 50 mL volumetric flask with a small amount of distilled water and 4 mL of the coloring agent; then, after 15 min of final volume adjustment, the absorbance was measured at 520 nm using a spectrophotometer. The physicochemical properties of the soil are presented in Table 3.

### 4.4. Statistical Analysis

Excel 2021 was used for the data calculation and preliminary analysis. IBM SPSS Statistics 20.0 software was employed to perform one-way analysis of variance (ANOVA) to analyze the significance of differences among various degradation levels. Pearson correlation analysis was used to describe the correlations between different indicators. Furthermore, we constructed a Partial Least Squares Path Model (PLS-PM) using the “innerplot” function in the R software package (R-4.4.1) “plspm” to study the direct and indirect effects of land use on N components. The model path was guided 1000 times to validate the estimated values of path coefficients (representing the direction and strength of linear relationships between variables) and explanatory variability (R^2^), and the model was evaluated using goodness of fit (GOF). Plotting was conducted with Origin 2021.

## 5. Conclusions

Vegetation degradation altered nitrogen distribution and storage in the alpine wet meadow and significantly reduced soil N availability, with significant changes in the concentrations of key soil N pools (TN, NO_3_^−^-N, NH_4_^+^-N, MBN). The impact of different degradation gradients on soil active organic nitrogen fractions varied significantly, with severe degradation exhibiting more pronounced effects on soil physicochemical and microbial properties. Additionally, vegetation degradation enhanced the activities of soil urease (URE) and nitrate reductase (NR), decreasing the activities of protease (PRO) and nitrite reductase (NiR), and also increasing the content of NO_3_^−^-N. The soil nitrogen transformation was influenced by a combination of physical, chemical, and biological processes. The Pearson correlation analysis revealed that soil temperature and moisture were significantly positively correlated with TN, MBN, NH_4_^+^-N, PRO, and NiR, and were significantly negatively correlated with NR and NO_3_^−^-N. Nitrogen components and nitrogen cycling enzyme activities were significantly positively correlated. The Partial Least Squares Path Model (PLS-PM) elucidated the relationships between soil properties and nitrogen components under different degradation levels, explaining 78% of the variance in nitrogen components. The degradation level, growing season, and soil physical properties had indirect associative effects on nitrogen components, whereas the soil enzyme activities exerted a direct positive influence on nitrogen components.

## Figures and Tables

**Table 1:** Interactions of vegetation degradation, the soil layer, and growth season. V represents vegetation degradation; S represents the soil layer depth; G represents the plant growing season; V × S represents the interaction between vegetation degradation and the soil layer; V × G represents the interaction between vegetation degradation and the growing season; and V × S × G represents the interactions among vegetation degradation, soil layer, and the growing season (*p* < 0.01).

Factor		TN g kg^−1^	NH_4_^+^-N mg kg^−1^	NO_3_^−^-N mg kg^−1^	MBN mg kg^−1^	URE mg g^−1^ 24 h^−1^	PRO mg g^−1^ 24 h^−1^	NR mg g^−1^ 24 h^−1^	NiR mg g^−1^ 24 h^−1^
V	F	81.03	65.10	59.11	46.24	2.28	8.30	32.25	45.45
	*p*	<0.01	<0.01	<0.01	<0.01	0.08	<0.01	<0.01	<0.01
S	F	21.70	70.11	13.98	71.67	11.55	31.43	31.07	38.75
	*p*	<0.01	<0.01	<0.01	<0.01	<0.01	<0.01	<0.01	<0.01
G	F	38.95	41.58	24.53	100.97	99.91	79.23	82.66	142.04
	*p*	<0.01	<0.01	<0.01	<0.01	<0.01	<0.01	<0.01	<0.01
V × S	F	6.81	1.82	0.64	1.63	1.69	4.37	3.89	7.83
	*p*	<0.01	0.10	0.70	0.14	0.13	<0.01	0.00	<0.01
V × G	F	8.55	2.56	11.25	3.92	5.43	7.57	8.01	8.08
	*p*	<0.01	<0.01	<0.01	<0.01	<0.01	<0.01	<0.01	<0.01
V × S × G	F	5.25	2.29	0.55	2.45	2.77	4.91	4.41	2.02
	*p*	<0.01	0.00	0.97	<0.01	<0.01	<0.01	<0.01	<0.01

**Table 2:** Basic characteristics of plants in different vegetation degradation levels of the wet meadow. CK: non-degraded vegetation; SD: slightly degraded vegetation; MD: moderately degraded vegetation; and HD: heavily degraded vegetation. The values were presented as the mean ± standard deviation. Capital letters indicate differences between different levels of degradation (*p* < 0.05).

Degradation Degree	Dominant Species	Coverage (%)	Height (cm)	Aboveground Biomass (g m^−2^)
CK	*Kobresia tibetica* + Potentilla anserine + Poa annua	96.25 ± 5.32 A	16.71 ± 2.98 A	355.90 ± 174.64 A
SD	*Carex* sp. *+ Potentilla anserine + Artemisia frigida* Willd + *Kobresia capilifolia*	86.34 ± 7.36 B	13.02 ± 2.24 B	293.02 ± 143.93 B
MD	*Artemisia frigida* Willd + *Artemisia frigida* willd + *Kobresia capilifolia*	45.33 ± 13.34 C	7.43 ± 0.97 C	185.73 ± 134.90 C
HD	The vegetation is sparse, with only a little *Artemisia frigida* Willd. and *Polygonum viviparum*			

**Table 3:** Physicochemical properties of soil in the experimental sites [18]. CK: non-degraded vegetation; SD: slightly degraded vegetation; MD: moderately degraded vegetation; HD: heavily degraded vegetation. pH: potential of hydrogen, BD: soil bulk density; SOM: soil organic matter; TP: total phosphorus; and TK: total potassium. The values are presented as the mean ± standard deviation. Capital letters indicate differences between different levels of degradation (*p* < 0.05).

Degradation Degree	pH	BD g cm^−3^	SOM g kg^−1^	TP g kg^−1^	TK g kg^−1^
CK	7.92 ± 0.04 A	0.36 ± 0.01 C	65.82 ± 13.64 A	1.48 ± 0.51 A	6.03 ± 0.41 A
SD	7.79 ± 0.06 B	0.39 ± 0.02 C	65.45 ± 9.67 A	1.29 ± 0.30 AB	6.02 ± 0.44 A
MD	7.77 ± 0.08 B	0.61 ± 0.05 A	54.39 ± 10.66 A	1.17 ± 0.08 B	5.74 ± 0.26 AB
HD	7.76 ± 0.06 B	0.56 ± 0.03 B	53.63 ± 10.66 A	1.15 ± 0.22 B	5.58 ± 0.42 B

## Data Availability

The datasets generated and analyzed during the present study are accessible from the corresponding author upon reasonable request.
